# Self-Care Behaviors in Pregnant Women with Gestational Diabetes

**DOI:** 10.31661/gmj.v13i.3235

**Published:** 2024-04-02

**Authors:** Malihe Rahimi, Hossein keikha, Rahil GhorbaniNia, Younes Azizi, Zahra Ovaisi, Shima Mohammadian, Faezeh Aminipour

**Affiliations:** ^1^ Reproductive Health Promotion Research Center, School of Nursing and Midwifery, Ahvaz Jundishapur University of Medical Sciences, Ahvaz, Iran; ^2^ Department of Nursing, School of Nursing and Midwifery, Zabol University of Medical Sciences, Zabol, Iran; ^3^ Noncommunicable Diseases Research Center, Bam University of Medical Sciences, Bam, Iran; ^4^ Faculty of Nursing, Kermanshah University of Medical Sciences, Kermanshah, Iran; ^5^ Maternal-Fetal Medicine Research Center, Shiraz University of Medical Sciences, Shiraz, Iran; ^6^ Gynecology Oncology Department, Kamali Teaching Hospital, Alborz University of medical sciences, Karaj, Iran; ^7^ Ilam University of Medical Sciences, Ilam, Iran

**Keywords:** Gestational Diabetes, Self-care, Lifestyle, Hyperglycemia

## Abstract

Gestational diabetes (GD) is a type of diabetes that is considered a prevalent health issue that has been reported to represent an increasing trend in the past two decades. It is demonstrated that the prevalence of GD is irrespective of the country’s income status and is associated with complications of maternal and fetal due to maternal hyperglycemia. Although no specific treatment for GD has been established, various studies have suggested self-care as a potential strategy. Therefore, the current study aimed to review the effectiveness of self-care in pregnant women with GD. The findings revealed that increasing awareness of the pathophysiology and consequences of GD along with improving self-care behaviors such as monitoring blood glucose levels, changing lifestyle, and maintaining mothers’ health can potentially improve indicators related to diabetes. However, further studies with larger sample sizes and evaluation of related clinical/laboratory indicators in this content appear to be crucially required to confirm current understanding.

## Introduction

Gestational diabetes (GD) is considered a prevalent health issue that has been reported to represent an increasing trend in the past two decades following the increment of diabetes prevalence [1][2][3]. GD, a type of diabetes that is primarily detected in pregnancy with an increase in blood glucose levels, is widespread irrespective of country’s income status and is frequently associated with complications of maternal and fetal due to maternal hyperglycemia. In fact, maternal hyperglycemia is described as one of the primary and major predictors of maternal-fetal complications and conflicting results have been reported regarding the effectiveness of HbA1c levels [4][5][6]. Preeclampsia and birth trauma are two main maternal complications related to GD, meanwhile, fetal complications associated with GD could include shoulder dystocia, macrosomia, intrauterine death, and stillbirth [7][8][9]. In addition, both the mother and newborn are prone to develop diabetes mellitus for the rest of their lives [10].

The growing incidence of GD has caused crucial concerns for the health system throughout the world [11][12]. However, it is widely suggested that interventions based on health empowerment are able to improve the glucose levels in the blood of pregnant mothers with GD in order to reduce the risk of mentioned complications [13][14]. Indeed, it has been evidenced by a variety of studies that monitoring blood glucose levels and modifications of lifestyle may overcome related complications [15][16]. For this purpose, females are required to be educated regarding diabetes-related self-care skills according to approved standards after diagnosis and according to their personalized requirements [17]. Although the suggested strategies represent the potency to improve the health outcomes of both mothers and newborns, they could be challenging for pregnant females with GD as these approaches require education and adopting skills related to self-care in a short period [18].

As a result, it appears to be necessary to determine the best approach that can achieve the lowest costs and requirements with the highest productivity and the most ideal output, which is the prevention of complications in the mother and fetus. For this purpose, the current study aimed to review the conducted investigations by focusing on a variety of domains including parental awareness of diabetes pathophysiology, blood sugar monitoring, healthy lifestyle, mental health of parents, and approaches based on smartphone applications. First of all, the current study represents a basic summary of the pathophysiology of GD, risk factors, preventive strategies, and current treatment approaches.

Overview of Gestational Diabetes

GD could be described as a metabolic disorder in which diabetes has occurred during pregnancy. It is reported that GD affects 14% of total pregnancies all around the world and corresponds to approximately 20 million births each year [19]. It is widely accepted that spontaneous hyperglycemia occurs during GD and major risk factors of the onset of GD include a parental history of diabetes, obesity, being overweight, a diet consisting of high fat and low carbohydrate, and a sedentary lifestyle [20][21]. In fact, valid sources such as the American Diabetes Association have defined GD as a metabolic disorder and a chronic state of glucose intolerance that corresponds to any degree of insulin resistance that occurs with the onset of pregnancy [22][23]. It is believed that GD represents no time-bound occurrence rate during the period of pregnancy, hence it could be triggered at any stage. Insufficient insulin secretion is considered the main reason for higher levels of blood glucose in patients with GD [24].

The most frequent symptoms of GD include elevated levels of thirst, dry mouth, repeated urination, and continuous fatigue [25]. Clinical investigations have revealed that the pathophysiological effects of GD are temporary, however, the aftereffects of GD on maternal and fetal health are prominently negative [25]. Indeed, it is widely accepted that GD is a temporary state in which its main symptoms are resolved when the period of pregnancy is over. Nevertheless, GD may cause a remarkable increase in the occurrence of metabolic complications including obesity, diabetes mellitus, and cardiovascular disorders. Unfortunately, no current medication is available to completely treat GD [26][27]. The oral glucose tolerance test (GTT) is considered a direct evaluation of an individual response to glucose intake that could effectively benefit the diagnosis of patients with GD as well as diabetes mellitus.

The fasting blood glucose (FBS) level at almost 105 mg/dl, and shifting of blood glucose levels from 190 mg/dl one hour postprandial to levels of 165 mg/dl two hours postprandial and 145 mg/dl three hours postprandial are main laboratory markers of GD. It has been widely demonstrated that FBS levels remain more than 126 mg/dl during the first trimester of pregnancy, the levels of glycated hemoglobin (HbA1c) remain at 6.5%, levels of random glucose are over 200 mg/dl, and levels of glucose two hours after a load of 75 g of oral glucose would be more than 200 mg/dL [28][29]. As mentioned earlier the pathoetiology of GD could be assumed to be related to metabolic complications such as obesity, insulin resistance, and dysregulation of other hormones and adipokines [30][28]. The hyperproduction of pro-inflammatory cytokines from the adipocytes that cause a chronic inflammatory state is considered the main reason for obesity’s contribution to GD incidence [31][32]. It has been revealed that inflammatory markers such as tumor necrosis factor (TNF), proinflammatory interleukins (IL), visfatin, and adipokines are actively secreted by adipocytes and/or immune cells as a result of established chronic inflammation [33][34], all of which reveals the close association between diabetes, obesity, and inflammation [35][36]. Indeed, the interplay between both proinflammatory and antiinflammatory cytokines during a healthy pregnancy preserves the metabolic homeostasis between the mother and fetus, however, in obese pregnant women, the higher secretion of proinflammatory cytokines is followed by metabolic dysregulation including insulin resistance and increased levels of glucose in both mother’s and fetal’s blood. Importantly, insulin resistance could be followed by elevated glycogenes (build-up of glucose), hyperglycemia, and hyperlipidemia that repeats the vicious cycle of obesity [37][38]. In addition, dysregulation of factors such as adiponectin, leptin, visfatin, sex hormone binding globulin, human placental lactogen, estrogen, progesterone, cortisol, and prolactin may play a role in the etiology of gestational diabetes through influencing insulin resistance, inflammation, atherogenesis, hypertension, food intake, and imbalance of glucose and fatty acid levels [39][40][41][28].

Self-Care Strategies During Gestational Diabetes

As discussed above, GD is a chronic and common condition that may occur due to metabolic, hormonal, and inflammatory disorders. Perhaps the complicated and multifactorial pathophysiological of the disease can be assumed as the most important reason that has led to the lack of complete treatment of GD. Nevertheless, early diagnosis and approaches based on self-care have been described as the two main strategies for confronting GD and preventing its consequences in both the mother and the fetus [42][43]. In general, self-care has been defined as processes related to establishing behaviors in order to ensure the holistic well-being of oneself, promote a healthy state of the person, and actively manage the disease as it occurs [44]. Self-care related to GD could be described as a set of actions that include increasing the level of health literacy, improving mental health, and considering effective procedures such as changing lifestyle and monitoring the symptoms of the disease [45]. A plethora of studies have suggested the effectiveness of self-care-based approaches to dealing with GD, however, each of them may represent limitations and strengths. According to the extent of investigations that have been conducted in this context, the present study has attempted to review the related studies in four separate chapters including the mother’s information regarding GD, blood glucose monitoring, healthy lifestyle, and parental mental health.

Parental Knowledge Information on GD

Previously, it has been determined that mothers must know about the pathophysiology, characteristics, and consequences of GD. In fact, knowledge of GD needs to consist of information about the definition, symptoms, causes, treatment, consequences, and complications of GD on maternal and fetal health [46]. The educational sessions could be delivered through a variety of teaching strategies such as lectures, prints, PowerPoint presentations, and videos. In addition to the teaching method, the duration of educational interventions has been mentioned as one of the influencing factors on clinical outcomes [47][1], which will be further discussed.

It has been suggested that patient education could be considered a major component of positive pregnancy and childbearing experiences, particularly in women with GD. A recent randomized controlled trial on 61 patients with GD who received a self-care education program with the usual care plan for GD revealed that implementing a tailored care education was able to improve clinical outcomes [48]. In fact, the researchers reported that self-care education along with the usual care plan for GD compared to the usual care plan resulted in a significant reduction in maternal and neonatal hospitalizations, preterm labor, cesarean section, fetal distress, macrosomia, newborn respiratory complications, and more importantly hypoglycemia [48]. Interestingly, a variety of studies have shown that self-care education can positively affect clinical outcomes and assist gestational diabetes management by reducing perceived stress, increasing health literacy, and improving self-care behaviors [49][50]. Moreover, it has been believed that self-care can be considered the first step to helping patients to manage their pathological condition more appropriately, therefore it is necessary for the patient to believe in his ability to do a task or adapt to a stressful situation, an ability that is known as self-efficacy. In fact, by examining 400 pregnant women with gestational diabetes, Kordi and colleagues have shown that self-belief can significantly improve the self-care behavior of patients [51].

In addition to the patient’s self-efficacy, determining the appropriate method of education may depend on other factors such as society’s culture, the patients’ spirituality and religion, the supportive role of spouse and family, etc [52].

On the other hand, the perfect pattern of self-care education for women with GD can be determined by evaluating the state of mental health, monitoring blood glucose, and observing a healthy lifestyle including adherence to physical activity, following a healthy diet, and considering waist circumference and body weight. In addition, a randomized controlled trial on 92 pregnant women with GD aimed to evaluate the effect of a four-session educational intervention, one session per week, consisting of usual prenatal care along with self-care education through lectures and question and answer. Moreover, an educational booklet was given to patients at the end of the first session. Interestingly, it was demonstrated that the intervention group had higher knowledge of the disease and self-care behaviors that resulted in improved impaired glucose tolerance [53]. Concordantly, one-to-one education has been assumed as a suitable approach for self-care education of pregnant women with GD, which can lead to the desired clinical outcomes [54]. Moreover, the possibility of using smartphone applications to educate women and follow up on self-care is suggested [13].

Glycemic Status Monitoring

Blood sugar monitoring can be included in the evaluation of FBS, 2-hour postprandial glucose (2hpp) levels, and the levels of HbA1C. A majority of the previous studies have considered the evaluation of FBS and 1h/2h postprandial glucose levels as a suitable approach to assess the status of pregnant women with GD [55][56][57]. However, conflicting results have been reported regarding the effectiveness of HbA1c levels evaluation [58][59].

Blood glucose monitoring could be considered one of the self-care behaviors that may be represented to women with GD as routine self-care training or through designed smartphone applications [13][54][53]. The efficacy of continuous blood glucose monitoring in improving the clinical outcomes of mothers and fetuses has been investigated by several studies. A randomized clinical trial involving 71 pregnant women with diabetes revealed that continuous glucose monitoring, as an educational tool to inform shared decision-making and future therapeutic changes at intervals of 4-6 weeks during pregnancy, could result in lower mean HbA1c levels, decreased mean birthweight, decreased birthweight centiles, and reduced risk of macrosomia [60]. In addition, the efficacy of continuous blood glucose monitoring and cost implications have been emphasized by other similar studies [61][62][63]. Concordantly, similar studies have emphasized the effectiveness of continuous blood glucose monitoring in improving diabetes-related laboratory parameters including FBS, A1c, 2hpp, etc., as well as clinical outcomes related to mother and fetal health complications [64][65][32]. Nevertheless, it appears that further large-scale investigations are required to confirm the most appropriate approach for continuous glucose monitoring in pregnant women with GD.

Healthy Lifestyle

A healthy lifestyle in women with GD can be described as consisting of two main components including a healthy diet/nutrition and appropriate physical activity [54][66][67]. Determining a healthy diet for diabetic mothers should include dos and don’ts regarding foods rich in carbohydrates, fats, and proteins. Whereas, none of the previous studies have introduced a specific index of the healthiness of a diet based on its nutritional composition, but have only considered generalities. On the other hand, the manner of mothers’ self-care education about a proper diet should be determined by further studies, because previous investigations do not support face-to-face follow-up of women with GD with a smartphone application in the presence of high-standard usual care [68].

Excessive gestational weight gain has been associated with adverse health outcomes in both the mother and newborn. Thereby, women are encouraged to be active physically before, during, and after pregnancy. A plethora of evidence is available that supports antenatal physical activity in order to bring health benefits for both the mother and child. In addition, lifestyle changes and behavior modifications are considered to be able to promote healthy postpartum weight loss and contribute to the prevention of GD [69][70][71]. A pilot study involving 30 overweight pregnant women revealed that health coach behavioral support can lead to encouraging mothers to participate in interventions to increase physical activity and observe healthy lifestyles [72]. However, a health coach does not appear to be available for all pregnant women with GD, especially those living in underdeveloped countries. Meanwhile, education related to self-care may lead to increased physical activity, regular exercise, and postpartum weight loss [73][74].

Mental Health

According to the definition provided by the World Health Organization, mental health is a state of mental well-being that makes a person capable of coping with the stresses that occur in life, realizing their personal abilities, learning and working well, and contributing appropriately to their community [75][76]. Mental health is considered an integral component of well-being that underpins individual and collective abilities in order to make decisions [75][76]. As mentioned earlier, the ability of pregnant women with gestational diabetes to be aware of the characteristics of the disease as well as learn self-care is dependent on self-belief. Self-belief can be considered one of the components of mental health, while other components are also considered effective. In this regard the state of depression, religious beliefs, positivity, belief in individual abilities, and family support can be considered other important aspects involved in mental health [49][51]. Considering that some of these aspects have been discussed previously in the "parental knowledge information on GD" section, the present study reviews other related investigations.

Horsch et al have examined the association between maternal mental health and the improvement of cardiometabolic complications in women with GD and their offspring [77]. In addition, Zandinava et al conducted a randomized controlled trial on 92 pregnant women with GD to evaluate the effect of educational package on self-care behavior, quality of life, FBS, and GTT. The results of their study demonstrated that self-care education for pregnant mothers with GD can improve the mental health and quality of life of patients, and more importantly, significantly improve diabetic indicators [53]. In addition, another study found that although blood sugar was not monitored by mothers with GD, self-care education, especially by health professionals, a positive attitude toward disease control, along with a healthy diet and regular exercise as self-care behaviors was able to affect blood glucose levels significantly [78]. On the other hand, postpartum depression in women who have had gestational diabetes leads to disrupting the lifestyle and can have unwanted results [79].

## Conclusion

The current review demonstrated that increasing the level of awareness of pregnant mothers with GD and educating them on self-care behaviors including monitoring blood glucose levels, changing lifestyle, and maintaining mental health can improve indicators related to blood glucose and possibly metabolic complications. However, the studies conducted appear to be preliminary, hence further research into this content is required.

## Conflict of Interest

None.
